# Signage as a tool for behavioral change: Direct and indirect routes to understanding the meaning of a sign

**DOI:** 10.1371/journal.pone.0182975

**Published:** 2017-08-30

**Authors:** Julia Meis, Yoshihisa Kashima

**Affiliations:** Melbourne School of Psychological Sciences, University of Melbourne, Victoria, Australia; Kyoto University, JAPAN

## Abstract

Signs, prompts, and symbols are a common means to change behavior in our society. Understanding the psychological mechanisms by which signage influences behavior is a critical first step to achieve the desired outcome. In the current research, we propose a theoretical model of sign-to-behavior process. The model suggests that when one encounters a sign, it is encoded to construct an action representation (comprehension process), which is then acted on unless its enactment is inhibited (decision process). We test the implications of the model in two studies. In support of our hypothesis, for unfamiliar signs, clarity of purpose predicts perceived effectiveness of a sign; however, for familiar signs, clarity of purpose does not matter. Insights gained from the studies will help to design effective signs. Practical implications of the model are discussed, and future research directions are outlined.

## Introduction

In our everyday life, we encounter many different kinds of signage. For instance, signs are used to give us directions (e.g., exit signs), control traffic on our streets (e.g., stop sign), facilitate the use of computer programs (e.g., save symbol), warn us about a potential danger (e.g., wet floor signs), or ask us to perform a specific behavior (e.g., turning off mobile phones). Signage plays an important role in our society as a means of transmitting a message in an attempt to persuade us what to do and what not to do, thus acting as stationary, persuasive communication [1]. Unlike mass media—an entity endlessly transmitting persuasive communications—signage does not rely on expensive high technology or a large number of people producing message contents. Furthermore, its recipients typically do not need specialized equipment either. So effectively every person who is equipped with sensory organs to perceive any signage is a potential recipient of the message whose behavior may be influenced. The relatively inexpensive maintenance cost of signage makes it a highly popular method for behavior change.

Although we are surrounded by signs in our everyday life, the underlying psychological process of behavior change through signage is not well understood. In the present paper, we will briefly review the evidence for the general effectiveness of signage and important sign characteristics first, propose a two-stage theoretical model that describes the psychological process from the encoding of a sign to behavior, and then report the results of two studies in support of the model.

### Effectiveness of signage

A body of research has shown that signs are effective in changing behavior in a variety of domains (e.g., road traffic [2–5], health behaviors [6–10], and environmental protection [11–20]). For example, in regard to road traffic—one domain heavily reliant on communication via signs—signs have been found to be successful in increasing safety belt usage [2], decreasing speeding [3], reducing deer-vehicle collisions [4], and reducing conflicts between pedestrian and motor vehicle drivers [5]. However, other domains have also benefited from the use of signage to initiate behavior change. For example, signs used in the health domain encouraged safer sex by promoting condoms [6], sun safety [7], correct lifting posture to prevent back injuries [8], stair use instead of elevator use [9], and protection against hearing damage [10]. In the domain of environmental protection, signs have been shown to be effective in a reduction of littering and an increase in recycling in a variety of settings, for example, in parking garages [11], football stadiums [12], cafeterias [13, 14], education and office environments [15, 16], as well as water and electricity use [17–20].

Moving beyond showing the effectiveness of specific sign(s) to affect behavior, other research has attempted to identify the characteristics of an effective signage. A prominent example is Geller’s work [21–28]. According to him [27], signs are more effective when (1) they are displayed in close proximity to the point of action of the requested behavior [21], (2) they specifically state what behavior is desired or describe alternative behaviors [22, 23], (3) the requested behavior is relatively convenient [24, 29, 30], and (4) the prompt is presented in a polite, non-demanding language [21, 22, 25]. Geller’s design principles still play an important role in the signage literature; they have been widely used to design prompts in signs (e.g., [17, 19]).

Although these principles provide a valuable starting point, there are limitations. First of all, whereas some principles have received empirical support, others have not. For instance, support is shown for principle (1) proximity [15, 18], and (3) convenience of the behavior [13]. However, empirical support for principle (2), specificity of the prompts, was found by some [15], but not by others [14]. Principle (4), polite, non-demanding language, has not received much support. Whereas Durdan et al.’s [14] findings show support for this claim, the studies by Reiter et al. [11], Baltes and Hayward [12], and Geller and colleagues [22] did not. For instance, Geller and colleagues [22] compared the effects of “Please don’t litter” messages versus “You must not litter” notes and did not find any differences in effectiveness, as well as, Reiter et al. [11], who achieved similar compliance rates by using “Pitch in!” and “Littering is unlawful and subject to a $10 fine” prompts.

Aside from the empirical limitations, there are two theoretical limitations. The majority of the above research has a relatively narrow research scope, i.e., they focus on anti-litter behavior [21–23, 25, 27]. It is an open question whether this framework can be extended to signage in other domains. Furthermore, Geller’s design recommendations are general guidelines for designing a signage, and they do not say how and why signs that follow these design principles may be effective. Thus, the existing literature has mainly focused on *‘whether’* and ‘*what sort of’* signs change behavior, but not so much on *‘how’*. In fact, there is little research on the underlying psychological mechanisms of how signage affects behavior.

#### Two-stage model of sign process

**Fig 1:**
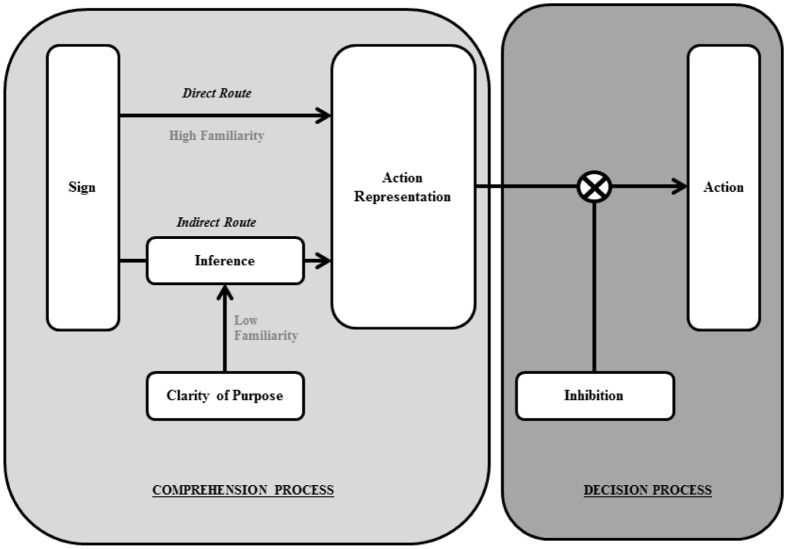
Schematic representation of the two-stage model of sign process.

In an attempt to advance our understanding of the psychological process underlying the effectiveness of signage, we propose a theoretical model of the sign-to-behavior process, discuss how it can shed light on the existing literature, and provide an initial test of the model. In particular, we suggest that two general processes are involved in the pathway from signage to behavior: comprehension and decision (see Fig 1). In the first comprehension process, we suggest that signage is perceived, its intent is understood, and an *action representation* is formed. By action representation, we mean a psychological representation of the action or the category of actions to be performed in a given context (to be explicated later). In the second decision process, a decision is made to act or not to act on the action representation. Following recent literature (e.g., [31, 32, 33]), we suggest that once an action representation for a well-learned behavior is constructed in the mind, the default is to act on it. Thus, unless there is some inhibitory process to stop the actor from acting on it, the action representation is likely to be carried out. Fig 1 schematically presents this model.

#### Action representation and decision process

Critical in our model is the concept of *action representation*. An action representation is a hierarchical structure that includes cognitive representations of a goal, sub-goals, and concrete actions that are typically performed to attain the goal and sub-goals given the context in which the action occurs (e.g., [34, 35]). For instance, the goal of having a sustainable lifestyle may include the sub-goals of reducing greenhouse gas emission, reducing water consumption, reducing waste production, etc. These sub-goals subsume more concrete actions that help the actor to attain them. So, the sub-goal of reducing greenhouse gas emission can be achieved by more concrete actions such as recycling more, riding a bicycle to work, eating less meat, wearing warm clothes in winter to reduce heating, etc. Thus, an action representation contains a hierarchical organization of goals, sub-goals, and behaviors.

We assume that a relevant action representation is already learned and accessible in most sign receivers’ minds. This is because signage typically targets well-learned behaviors that most sign receivers know how to perform. Examples include such behaviors as anti-littering [27], safety belt use [2], recycling [16, 22], turning off taps or lights [17–19], and the like; signage is not designed to teach new behavior skills. In other words, signs are designed to prompt people to access the relevant action representation that they already possess and activate it at the right time in the right context.

In the sign-to-behavior process, we propose that, when actors see a sign, they activate an action representation of the sort described above. So, for instance, when actors see a sign to save water, they activate an action representation of saving water in their minds. This action representation is hierarchically organized, so the perceived sign can lead to the representations of reducing water in many different areas in everyday life, such as having shorter showers, less plant watering, turning off the tap when washing the dishes, or brushing teeth. In other words, perceiving a sign and understanding its intent is likely to result in the activation of an action representation including information about the activated goal (i.e., saving water), sub-goals (i.e., saving water in the bathroom, the garden, and kitchen), and concrete behavioral steps to achieve what the sign requests (i.e., having shorter showers, watering the garden less, not doing the dishes under running water).

In line with this, there is evidence for the importance of action representation in the sign-to-behavior literature. Recall that Geller’s principle to specify an action to be performed directly speaks to this. A range of studies have shown the effectiveness of a sign that clearly specifies what to do [22, 23]. An exception is Durdan et al.’s [14] work, which prompted cafeteria patrons to clear their table after use. In their study, a general prompt to clear their table and a specific prompt to return their tray and dishes in the tray holders did not show any differences in effectiveness. Our model suggests that this was because the cafeteria patrons had a well-developed action representation that contains a goal (i.e., clear table) and a specific action (i.e., return trays and dishes to tray holder). Just by prompting the goal, the specific action representation was already activated and therefore performed. Likewise, Aronson’s work [17] shows the importance of linking a goal to a specific action. In this study, patrons were asked to turn off the shower while soaping. The research showed that the sign was effective when the link between the goal to reduce energy consumption and the specific action requested was existing, i.e., turning off the shower while soaping. In order to reduce the energy consumption (goal), the consumption of warm water needs to be reduced (sub-goal); in order to reduce the consumption of warm water, the shower needs to be turned off when unnecessary. Thus, the present model can shed light on some of the findings for which Geller’s principles cannot provide adequate explanations.

Recent research suggests that, once an action representation for a well-learned behavior is formed, the actor is highly likely to perform the behavior unless its enactment is inhibited ([e.g., [31, 33]). Evidence for this comes from research on motor action and imitation. For instance, when a person is interacting with another person, the former tends to mimic the latter’s nonverbal behaviors (e.g., rubbing face, touching hair) without awareness (e.g., [36]). Likewise, an individual’s action performance is facilitated by the observation of another person’s similar action, but inhibited by the observation of a different action (e.g., [37, 38, 39]). Therefore, an action representation for a well-learned behavior (such as those typically implicated by signage e.g., turn off the lights, save water, do not run), once activated, is likely to energize the actor to perform it.

This tendency to perform an action congruent with an action representation can be inhibited when there is a need and an appropriate psychological resource to do so (e.g., [39, 40]). In the context of sign-to-behavior process, two main mechanisms have been suggested. One is the issue of convenience [27]. If an action requested by a sign is inconvenient, the perceived cost of enacting it may inhibit performing the action. For instance, prompts to pick up someone else’s litter were not found to be effective, unless the litter negatively influenced an individual’s aesthetic preference [24, 29, 30]. Some research suggests that the use of threatening language might result in an inhibition via psychological reactance [14] although other studies have found that politeness was not a major determinant of a sign’s effectiveness as we noted earlier [11, 12, 22].

There is a rather counter-intuitive finding that the current model may help to understand [22]. In this study, prompts explicitly stating to litter (“Please litter. Dispose of on the floor”) generated more litter on the floor than a baseline condition without prompts! Were they “obeying” the sign’s prompt? Rather, this may be interpreted as people unthinkingly performing the behavior when they activated the action representation to litter even though a moment’s reflection would have inhibited this action.

#### Comprehension process

The foregoing discussion implies that it is critical to examine how a sign is transformed into an action representation. This is what we call a comprehension process. Past research on signage suggests that, depending on the type of signage, its comprehension requires different competences [41, 42]. *Symbols* are one type of signage, which mainly relinquishes the use of words, and instead work with graphic features (e.g., floppy disk on user interfaces). They require the perceiver to know the meaning of the graphic symbol. *Prompts* on the other hand rely mainly on language and thus the ability of the perceiver to comprehend what the prompt asks for (e.g., “STOP” sign at street corner). Lastly, *signs*–which can be seen as a combination of symbols and prompts—use both linguistic information and graphical features to transmit a message. The aim of symbols, prompts, and signs is to transmit a message about what to do or what not to do, and as such it is akin to a type of linguistic communication called a speech act, particularly, what Searle [43] called *directives*. Just as a prompt, “Turn off the light!” is a directive, the symbol that shows a finger flicking the light switch is a directive, thus they both can be seen as an attempt by the sign maker to get the viewer to perform a specific action. In this sense, the cultural function of symbols, prompts, and signs is much the same; for this reason, we use the term sign, to refer to them all in this paper.

Signs as a device for communication can also be considered as a means of changing behavior. To the extent that signs can be thought of as something akin to a type of speech acts called directives [43], a conceptual analysis of speech act should be able to shed further light on the process by which signs influence human behaviors. Directives are defined as an attempt by the speaker to get the hearer to perform a specific action [43]. There are two processes by which linguistic speech can have an effect on its receiver. The first route is the *direct* understanding of the literal meaning, that is to say, *‘what is said is what is meant’*. So, “Please recycle” can be directly understood as a direction for the hearer to recycle his or her rubbish, when the speaker and receiver are familiar with the same set of contextual background assumptions [44]. In this case, the receiver has to be familiar with the practices of recycling in the given context. This might sound obvious, but it is worth remembering that recycling practices differ between countries and sometimes even between different regions within countries.

The second route is *indirect*. In contrast to the direct route, there is no explicit correspondence between what is said and what is meant. Take the example of someone saying to you, “There is a recycling bin in the room.” If you take the direct route as outlined above, you would interpret this utterance as information that there is a recycling bin in the room. However, you would not come to this interpretation under most circumstances, but would infer that the speaker is directing you to place recyclables in the recycling bin. In this case, the speaker is *not* saying what he *means*, but what he is saying *implies* what he means [44]. Therefore, the listener must *infer* what the speaker means. The critical point of the indirect route is that the receiver has to infer correctly the course of action that the speaker is requesting [for further examples see 43, 45, 46]. The ease with which these inferences can be drawn depends on the clarity of the message.

In the case of signage, too, the direct and indirect routes can both occur; just as in linguistic speech acts, the likelihood that one or the other occurs depends on the extent to which the sign receiver is familiar with the sign. If a viewer has seen a sign many times before—thus highly familiar with the sign—a direct route to comprehension is likely to occur. The viewer has had many occasions to process the information presented in the sign and know its meaning, and therefore the sign is directly translated into an action representation. A good example is Gary Anderson’s well-known sign of recycling—the three chasing arrows (often in green) that can be found on many recyclable items. Most people in industrialized parts of the world are so familiar with it that it acts as a direct reminder of recycling and an action representation is likely formed which says, “This item is recyclable. Throw it into the recycling bin”. However, imagine someone from a culture that does not use this sign (i.e., low familiarity). In this case, this actor is not able to activate an action representation via the direct route, as he or she is not familiar with the sign. Consequently, the indirect route needs to be used, which means the actor needs to *infer* the intended meaning. Nonetheless, because its intended meaning is fairly opaque, it would be well-nigh impossible to guess what it means and follow its directive about what to do. For the actor who is not familiar with a sign, the sign’s meaning (i.e., what it directs the actor to do or not to do) must be obvious. Therefore, in the case of unfamiliar signs, their effectiveness must depend on the extent to which the purpose of the sign can be unambiguously inferred.

### Present studies

Thus, there are two routes in the comprehension process to an appropriate action representation based on a sign. First, if the sign is familiar, it takes the direct route. The viewer of the sign will directly “read off” the intended meaning. However, if the sign is unfamiliar, the viewer will need to “infer” the intended meaning to activate an appropriate action representation. To infer the appropriate action representation via the indirect route, however, clarity of a message’s intent is critical. This analysis provides a testable hypothesis about a sign’s effectiveness. There should be an interaction effect between familiarity of a sign and clarity of purpose discernible from the sign on the sign’s effectiveness. If signs are unfamiliar, clarity of purpose should predict effectiveness; however, if they are familiar, clarity of purpose should not matter. We test this hypothesis in the studies reported below and thus will focus on the comprehension process.

In so doing, the present paper also describes a new, practical method by which the effectiveness of signs can be evaluated. As reviewed earlier, the existing literature on sign effectiveness typically evaluates a sign’s effectiveness one by one. For instance, in order to evaluate the effectiveness of a recycling sign, the actual behavior of recycling is measured when the sign is displayed near a recycling bin when compared to when it is not (e.g., [15]). Whereas this method has an advantage of being able to observe the impact of a sign on an actual behavior, it is very costly to evaluate every sign this way when there are many possible signs to choose from. We describe a method by which the effectiveness of a large number of signs may be evaluated and its significant determinant may be identified. In particular, we use *perceived effectiveness* to index the effectiveness of signs.

Perceived effectiveness has been used to examine a range of behavior change issues, including promotion of hand hygiene [47], pregnancy-related cigarette package health warnings [48], physical activity apps [49], image of green products [50], feeling of connectedness [51], traffic control [52], etc. However, there are only several scholars that investigated the link between perceived effectiveness and actual effectiveness. Dillard and colleagues [53] showed support that perceived message effectiveness predicted post-message attitude and behavioral intention in regard to various topics ranging from flossing, alcohol consumption, seat belt use to public service announcements on dangers of drugs, risky sex, drunk driving, cigarettes, and television. One important work in regard to perceived effectiveness is the work by Brennan, Durkin, Wakefield, and Kashima [54], which showed that people’s perception of effectiveness of a quit smoking TV advertisement predicted actual effectiveness of this ad in a laboratory setting. Recently, Davis et al. [55] replicated those findings with survey data of a population-based representative samples of smokers, showing again that higher perceived effectiveness was associated with increased odds of quit attempt at follow up. Since then, perceived message effectiveness as an indicator of behavior change has also been shown in obesity research [56]. We will thus use perceived effectiveness to evaluate the effectiveness of signs in this research.

## Study 1

### Method

#### Participants

Fifty undergraduate psychology students (40 females; 2 did not indicate their gender) took part in this study for course credit. Age ranged from 18 to 45 (*M* = 20.04*; SD* = 5.02). Participants read a plain language statement informing about the research and checked a consent box as written consent before being allowed to participate. This study was approved by the Department Human Ethics Advisory Group, Melbourne School of Psychological Sciences, University of Melbourne (#1544720.1).

#### Materials

A total of 51 signs were used for this study. Half of the signs were categorized as environmental signs and the other half as non-environmental signs. Environmental signs covered the following different topics: recycling (5 signs; e.g., “Reuse, Reduce, Recycle”), paper use (5 signs; e.g., “Think before you print”), water use (6 signs; e.g., “Save water”), electricity use (5 signs; e.g., “Switch off”), and sustainable transport (5 signs; e.g., “I get around”). Non-environmental signs were also taken from five different areas: safe community living (5 signs; e.g., “Don’t leave your luggage unattended”), hospital (5 signs; e.g., “Red cross”), emergency (5 signs; “Police”), marine traffic (5 signs; e.g., “No windsurfing”), and construction (5 signs; e.g., “Construction site keep out”). A written description of the signs (S1 Table) and descriptive statistics for each group of signs (S2 and S3 Tables) can be found in the supplementary materials.

#### Procedure and measures

Study 1 and 2 were programmed and presented using Qualtrics software and participants took part in this 30 minutes experiment online. After being introduced to the study, participants were presented with 35 signs in random order (Qualtrics randomization function was used). Each sign was presented for five seconds before participants answered three questions. The first question was designed to tap Familiarity, and asked, whether they had seen the sign before. Answers were given on a scale from 0 *(never)* to 3 *(yes*, *often)*. Question 2 was used to measure Clarity of Purpose, by asking what participants thought the purpose of the sign was (asked to select one of the following four options: “Environmentally friendly behavior”; “A safe community living”; “Health and first aid information”; “None of the above”). Question 3 measured Perceived Effectiveness by asking how likely the sign would influence their behavior on a scale from 0 *(not likely at all)* to 100 *(very likely)*. The relevant sign was in view while answering the questions.

Our approach here was to use *signs* rather than participants as a unit of analysis, and so the number of signs represents the sample size (*n* = 51). We measured the extent to which each sign was (1) familiar to the current sample of respondents (Question 1: Familiarity), (2) able to clearly convey the specific action it recommended to do (or not to do; Question 2: Clarity of Purpose), and (3) seen to be effective in influencing behavior (Question 3: Perceived Effectiveness). The means of responses for Questions 1 and 3 were calculated for each sign to measure the sign’s Familiarity and Perceived Effectiveness. The use of our outcome measure builds on findings in the health communication. As we noted earlier, perceived message effectiveness has been shown to be a valid indicator of post-message attitudes and intentions [53]. In particular results by Brennan, Durkin, Wakefield, and Kashima [54] showed that perceived effectiveness of a quit smoking TV advertisement was a valid predictor of actual effectiveness of this ad, which was also supported by Davis and colleagues [55] work. Perceived message effectiveness was an indicator of behavior change in obesity research as well [56]. As our study looks at the effectiveness of a message to change behavior, we suggest that perceived effectiveness of a sign to influence behavior is a good proxy measure of actual effectiveness.

In order to measure a sign’s Clarity of Purpose, we used Question 2 to calculate the variability of responses for a categorical variable, a statistic analogous to a variance, following Kader and Perry [57]. In their article, Kader and Perry [57] described a “coefficient of unalikeability” (*u*2). The formula for the unalikeability coefficient for a categorical variable is as follows:

$$u_{2}=1-\overset{m}{\underset{i=1}{\sum }}{\left(\frac{k_{i}}{n}\right)}^{2}$$

where n is the number of participants evaluating a particular sign, m is the number of options in a categorical variable (4 in the present case), and k_i_ is the number of participants who selected the i-th option.

This coefficient varies between 0 and 1 with 0 indicating that the respondents’ answers were identical (i.e., no variability) and 1 indicating that the respondents’ answers are maximally diverse. With regard to Question 2, it indexes the variability in inferences the respondents made about the purpose of a sign. If the purpose of a sign is clear (i.e., high Clarity of Purpose), most of the respondents should make one and the same inference, and the index of unalikeability, *u2*, close to 0. Participants could choose from four different purpose options for each single sign. If a sign (e.g., universal recycling sign with three arrows) has a very clear purpose, most of the respondents would indicate the same option for this sign (i.e., environmentally friendly behavior). Thus, the variance of this categorical variable would be very low, as most of the respondents indicate the same option. If, however, the sign does not communicate a clear purpose, some respondents may indicate its purpose as “environmentally friendly behavior” whereas others may choose “a safe community living” or some other option, for instance. The more people differ in their judgement about the purpose (thus, the more variation in their answer pattern in regard to option 1, 2, 3, or 4) the higher the coefficient of unalikeability. To compute the coefficient of unalikeability, we counted the number of responses for each Clarity of Purpose option per sign, as well as how many participants evaluated the sign in total. This information allowed us to calculate *u*_2_. As we were not interested in how often observations differ from one another, but instead how often participants selected the same option as the purpose of a sign, we computed 1- *u*_2_, which ranged from 0 to 1, with 0 indicating that participants had chosen maximally divergent options (i.e., low Clarity of Purpose), and 1 indicating that they all had chosen the same option (i.e., high Clarity of Purpose). Higher coefficient scores thus were interpreted as more clarity about the sign’s purpose (e.g., less unalikeable), whereas lower scores were an indicator for a less clear message of the sign.

### Results and discussion

We tested the hypothesis that Clarity of Purpose is a significant predictor of Perceived Effectiveness of signs. Additionally, Familiarity is expected to moderate the effect of Clarity of Purpose on Perceived Effectiveness. This implies that Familiarity and Clarity of Purpose would have an interaction effect. To test an interaction effect, we followed a procedure recommended by Aiken and colleagues [58]. First, Clarity of Purpose and Familiarity were mean centered because this operation reduces the correlation between the main effect terms and the interaction term, thereby avoiding the problem of multicolinearity. The mean centered variables were then entered into a hierarchical multiple regression analysis. In a first step, the two variables Clarity of Purpose and Familiarity were entered and results revealed that the model accounted for a significant amount in Perceived Effectiveness, *F*(2, 48) = 16.69, *p* < .001. Thus, 41% of the variance in Perceived Effectiveness was explained by Clarity of Purpose and Familiarity (*R*^2^ = .41). In a second step, the interaction between those variables (Clarity of Purpose x Familiarity) were added to the regression model and this significantly increased the amount of variance accounted for, Δ*R*^2^ = .10, *F*(1, 47) = 9.85, *p* < .01. Δ*R*^2^ –representing the increase in explained variance—was 10%, thus adding the interaction term to the first model increased the explained variance by 10%. The F-test shows that this increment was statistically significantly greater than zero, suggesting the importance of the interaction effect. The total variance explained by the model as a whole was 51.2%, *F*(3, 47) = 16.46, *p* < .001.

**Fig 2:**
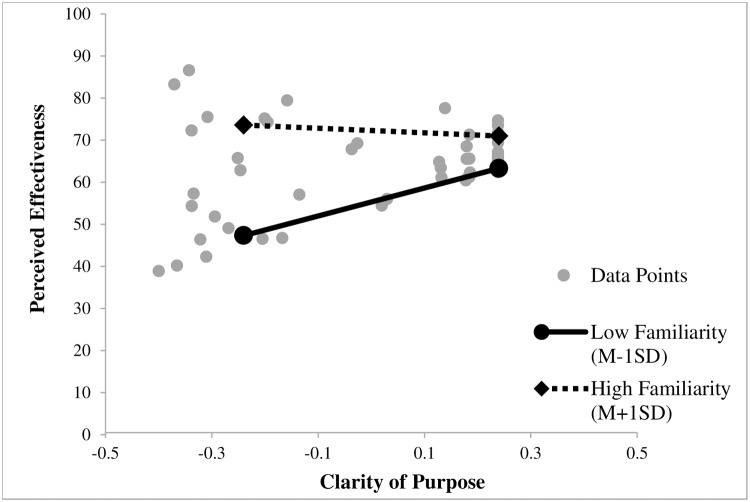
Moderation effect of the variable familiarity on perceived effectiveness displayed for clarity of purpose at low (*M* − 1*SD*) and high (*M* + 1*SD*) values of the predictor and moderator for Study 1. Clarity of Purpose and Familiarity are mean centered.

Examination of the interaction plot (see Fig 2) with mean centered variables showed patterns consistent with our hypotheses. For familiar signs, Clarity of Purpose does not influence Perceived Effectiveness of the sign. Simple slope analysis did not reach statistical significance for a Familiarity value of one standard deviation above the mean (*b*_*high*_ = -5.44, *t* = -0.62, *p* = .54). However, for signs that are not familiar, Clarity of Purpose impacts Perceived Effectiveness of a sign. Simple slopes analysis for a Familiarity value of one standard deviation below the mean was significant (*b*_*low*_ = 33.28, *t* = 3.73, *p* < .001). Table 1 displays the regression coefficients.

**Table 1:** Descriptive statistics and unstandardized regression coefficients for Study 1.

	*SD*	*B*	*SE B*	*t*	*p*
Constant		63.81 [60.80, 66.81]	1.49	42.71	*p <* .001
Clarity of Purpose (mean centered)	.24	13.92 [1.20, 26.64]	6.32	2.20	*p <* .05
Familiarity (mean centered)	.91	9.33 [5.98, 12.69]	1.67	5.60	*p <* .001
Clarity of Purpose x Familiarity	.23	-21.27 [-33.91, -7.64]	6.78	-3.14	*p <* .01

Note: Clarity of Purpose and Familiarity were mean centered (i.e., raw score—mean), and therefore the mean of those variables is zero.

## Study 2

### Method

Study 2 was designed to expand and replicate the findings of Study 1 by increasing the number of participants and signs presented.

#### Participants

Ninety-one participants (57 females, 7 did not indicate their gender) took part in the experiment for course credit. Age ranged from 18 to 48 (*M* = 20.07, *SD* = 4.83). Prior to participation, participants were informed about the study in a plain language statement and indicated their consent. The study was approved by the Department Human Ethics Advisory Group, Melbourne School of Psychological Sciences, University of Melbourne (#1545446.1).

#### Materials

Twenty signs were added to those from Study 1, resulting in a total of 71 signs (*n* = 71). The new signs were taken from the following areas: marine wildlife (5 signs; e.g., “Danger—Seals bite!”), wildlife (5 signs; “Do not touch animals”), additional safety signs (5 signs; “Do not run”), and additional environmental signs (5 signs; “Do not waste water”).

### Procedure and measures

Participants took part in Study 2 online. Study 2 consisted of two parts taking 60 minutes to complete in total. Part 1 took 30 minutes and was identical to Study 1. Only data from Part 1 will be reported here. Part 2 was for other tasks unrelated to the present research, and therefore will not be reported. The procedure and measures of Part 1 were similar to Study 1 with the exception that Clarity of Purpose was measured with a question that listed eight response options about the purpose of a sign. In addition to the four options from Study 1 (i.e., “Environmentally friendly behavior”; “Safe community living”; “Health and first aid information”; “None of the above”), the new options were: “Construction zones”, “Marine traffic”, “Emergency services”, and “Wildlife”. The redefinition of this question ensured that each sign could be assigned to an appropriate category. Additionally, each sign was evaluated by a minimum of 45 participants and a maximum of 50 participants. Each participant was presented to a random sample of 35 signs using Qualtrics randomization function.

### Results and discussion

In Study 2, we tested the same hypothesis using the same analysis as in Study 1. The first step of the hierarchical multiple regression revealed that the model with the two variables Clarity of Purpose and Familiarity accounted for a significant amount of the outcome variable, *R*^2^ = 0.33, *F*(2, 68) = 16.34, *p* < .001. Accordingly, the model explained 33% of the variance in Perceived Effectiveness. The interaction term Clarity of Purpose x Familiarity entered in a second step accounted for an additional significant amount of variance, *ΔR*^*2*^ = .07, *F*(1, 67) = 7.99, *p* < .01 and indicated a moderation effect. The total variance explained by the model (*R*^2^) as a whole was 40%, *F*(3, 67) = 14.68, *p* < .001.

**Fig 3:**
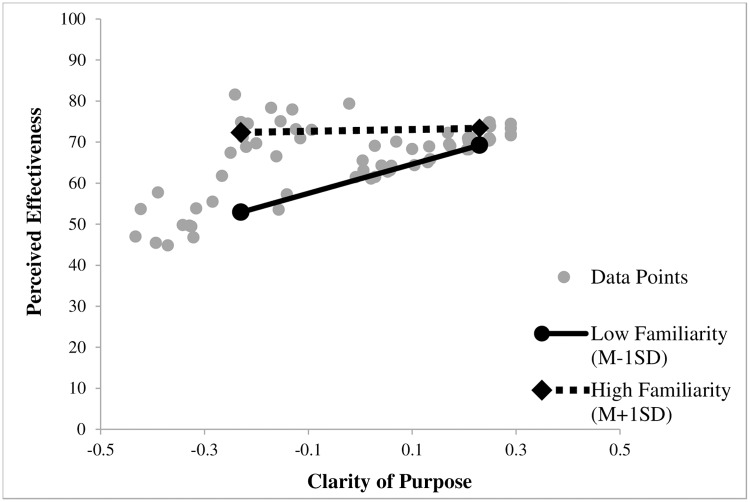
Moderation effect of the variable familiarity on perceived effectiveness displayed for clarity of purpose at low (*M* − 1*SD*), and high (*M* + 1*SD*) values of the predictor and moderator for Study 2. Clarity of Purpose and Familiarity are mean centered.

The interaction plot with the centered variables (see Fig 3) revealed a similar interaction pattern as in Study 1 showing a moderating role of Familiarity on Clarity of Purpose. Simple slope analysis was significant for low values of Familiarity (*M* − 1*SD*), *b*_low_ = 35.53, *t* = 4.53, *p* < .01, but did not reach significance for high values of Familiarity (*M* + 1*SD)*, *b*_*high*_ = 2.15, *t* = 0.25, *p* = .81. Table 2 shows the regression coefficients.

**Table 2:** Descriptive statistics and unstandardized regression coefficients for Study 2.

	*SD*	*B*	*SE B*	*t*	*p*
Constant		66.95 [64.34, 69.56]	1.31	51.22	*p <* .001
Clarity of Purpose (mean centered)	.23	18.84 [7.17, 30.52]	5.85	3.22	*p* < .01
Familiarity (mean centered)	.89	6.62 [3.67, 9.58]	1.48	4.47	*p <* .001
Clarity of Purpose x Familiarity	.20	-18.75 [-31.99, -5.51]	6.63	-2.83	*p* < .01

Note: Clarity of Purpose and Familiarity were mean centered (i.e., raw score—mean), and therefore the mean of those variables is zero.

## General discussion

The present paper has extended the existing literature on the effectiveness of signs in two main respects. First, we proposed a theoretical model through which to conceptualize psychological mechanisms underlying the sign-to-behavior process, and reported two studies to provide a preliminary test of the first part of the model. Second, in so doing, we have described a practical method by which to evaluate the effectiveness of a large number of signs and investigate its critical ingredient, i.e., clarity of purpose. We will discuss these points in turn.

Theoretically, we conceptualized signs as something akin to what speech act theory calls directives, and investigated two routes to the generation of an action representation. The first route refers to a direct understanding of signs due to high sign familiarity, and the second route is an indirect understanding via inferring the sign’s intent correctly due to a clear purpose of the sign. Consistent with this analysis, we showed the interaction effect between familiarity of a sign and clarity of the sign’s purpose on perceived effectiveness of signs. For familiar signs, clarity of purpose did not influence perceived effectiveness. Thus, for very well-known signs, like for example the universal recycling sign, perceived effectiveness is independent of clarity of purpose. The viewer processes the information via the direct route. For an unfamiliar sign, clarity of purpose played a significant role: The more clearly the purpose of the sign can be inferred, the greater was the perceived effectiveness of the sign.

Second, these findings give credence to the proposed method by which to evaluate the effectiveness of a large number of signs. Note that the existing literature of sign effectiveness tends to examine the effect of one specific sign on people’s actual behavior. Whereas this method permits the evaluation of a single sign’s effect on actual behavior, it is extremely costly to evaluate the effectiveness of a large number of signs if each sign has to be evaluated this way. Instead, we used a different approach of examining multiple signs and *perceived* effectiveness of signs as an outcome measure. The method allows us to investigate the effectiveness of a large number of signs in a single study. In addition, the current findings suggest that “Clarity of Purpose” of a sign as we measured it based on the coefficient of unalikability is useful in investigating a determinant of a sign.

Despite these strengths, the present investigation has a number of limitations. To begin, the proposed theoretical framework can be investigated by examining the comprehension and decision processes more thoroughly. For instance, we only showed that the Clarity of Purpose as measured in terms of the variability in viewers’ inferences about the meaning of a sign is a critical factor. Additionally, viewers had a relatively high level of education; viewers with other educational levels (particularly lower levels) may have shown different patterns of understanding. However, as others have noted [19, 59], in order for a sign to be effective, it needs to be attended to first, before its intent is inferred. Our findings only speak to the importance of the encoding of a sign’s intent. Furthermore, our research did not investigate under what circumstances an action representation may be translated into an actual action, and when this representation-action process is inhibited. Clearly, the decision process needs to be further investigated.

This consideration relates to the second limitation of the research. Although previous research by Brennan, Durkin, Wakefield, and Kashima [54] showed the use of perceived effectiveness as a useful index of actual effectiveness of a message in the health domain, future work should investigate its applicability using observed behavior as a dependent variable. Future studies can be conducted in lab and field settings to test for ecological validity. In the lab environment, signs with varying degrees in familiarity and clarity could be used to measure participants’ compliance with those specific signs. Observing behaviors like recycling, turning off lights after leaving the lab, closing the door, not using mobile phones, only talking softly with a confederate, not drinking or eating in the laboratory setting could be used as alternative outcome measures. Sign variations in regard to high or low familiarity and clarity should be pretested using a similar approach as described in Study 1 and 2 and measured in regard to perceived effectiveness. Studies of this kind would provide information about the implications of various degrees of familiarity and clarity on actual behavior and thus permits an examination of the decision process. Furthermore, by measuring actual behavior compliance and perceived effectiveness in a between subjects design, the evaluation of perceived effectiveness as a valid predictor for actual behavior can be estimated.

In addition, future work can investigate sign compliance in people’s intact behavioral settings. Potential studies could measure people’s compliance to signs in the environment in which they ordinarily live (e.g., university complex, company building, factory, sports facilities). In doing so, it would be recommended to work with people from diverse educational backgrounds. Signs can be varied in regard to the two critical components, familiarity and clarity, and participants’ behavior may be observed in field experiments. Future studies will be able to shed further light on the accuracy of perceived effectiveness as a predictor for actual behavior, assess actual behavior change, and investigate the influence of the built environment on sign effectiveness.

The current theoretical framework and findings have practical implications. First, our perspective advises sign designers and implementers that they need to pay close attention to familiarity and clarity of a sign. In particular, in designing *new* signs, emphasis should be placed on the clarity of the transmitted message and purpose of the sign. This is because people would not be familiar with those new signs, and therefore the clarity of the purpose of those signs is critical. The method used in this paper can be used to evaluate those new signs. For established signs, reassessing the clarity of purpose in those signs may become increasingly more important in a highly diverse world with high rates of immigration and travel. Established signs may be highly familiar to the local residents, but may be difficult to understand for foreign visitors, new immigrants, and more generally recent arrivals to a local area. To communicate effectively to a wide range of people, an ideal sign should be highly familiar to facilitate its direct processing, but at the same time, highly clear and unambiguous in its intended message (i.e., what to do and what not to do). This knowledge can help government agencies (e.g., Department of Transport, Department of the Environment, Department of Health), as well as the private sector (e.g., companies, factories, leisure time facilities) to change behavior effectively to reduce the risks of accidents, injuries, pollutions, etc. In conclusion, because signs are an important and inexpensive method for changing behavior, understanding the underlying mechanisms in behavior change through signage is fundamental for effective signage in the future.

## Supporting information

S1 Table Signs for Study 1 and 2. (DOCX)

S2 Table Descriptive statistics for perceived effectiveness, familiarity, and clarity of purpose for Study 1. (DOCX)

S3 Table Descriptive statistics for perceived effectiveness, familiarity, and clarity of purpose for Study 2. (DOCX)

S1 Text Assumptions of multiple regression. (DOCX)
