# Supplementary material for: Signage as a tool for behavioral change: Direct and indirect routes to understanding the meaning of a sign
Source: PLoS One. 2017 Aug 30;12(8):e0182975. doi: 10.1371/journal.pone.0182975 (PMC5576639; doi:10.1371/journal.pone.0182975)
Supplement: S1 Table — (DOCX) [file pone.0182975.s001.docx]

# S1 Table. Signs for Study 1 and 2.

## Environmental Signs

### **Reuse / Recycle**

| **Sign Number** | **Sign Description** |
| --- | --- |
| 1 | The University of Melbourne  Mixed Recycling  No plastic bags |
| 2 | Keep cups  There is enough plastic in 28 disposable cups and lids to make this keepcup |
| 3 | The University of Melbourne  Refill not landfill  Bring your own bottle to reuse. |
| 4 | Universal recycle sign |
| 5 | Reuse, reduce, recycle, plus unviersal recycling sign in upper right corner |

### Paper Use

| **Sign Number** | **Sign Description** |
| --- | --- |
| 6 | The University of Melbourne  Two sides are better than one.  Print double sided. |
| 7 | The University of Melbourne  One-sided paper for reuse |
| 8 | Think before you print |
| 9 | Remember these come from trees.  This sticker will save up to 100lbs of paper every year. Help spread the word. Get yours at: TheseComeFromTrees.com |
| 10 | Recycle Paper  Universal recycle sign |

### Water use

| **Sign Number** | **Sign Description** |
| --- | --- |
| 11 | The University of Melbourne  Save water.  Report any leaks to property & campus services |
| 12 | Water drop:  Save water |
| 13 | Every drop counts |
| 14 | Think! Conserve Water |
| 15 | Water drop hold in a hand (no text) |
| 16 | Turn off the tap while brushing teeth, soaping hands and shaving. |

### Energy Use

| **Sign Number** | **Sign Description** |
| --- | --- |
| 17 | The Unviersity of Melbourne  Switch off reduce our carbon footprint |
| 18 | The Unviersity of Melbourne  We need rest too.  Turn off monitors. |
| 19 | The Unviersity of Melbourne  Switch off monitors and computers to reduce our carbon footprint |
| 20 | Please turn off lights when not in use. |
| 21 | Burn calories not electriciy  Take the stairs! |

### Green Transport

| **Sign Number** | **Sign Description** |
| --- | --- |
| 22 | The University of Melbourne  Easy to get to from any direction.  Walk. Ride. Bus. Tram. Train |
| 23 | A better way to campus. Sustainable Transport.  Bus. Train. Tram. Car share. Walk. Ride. |
| 24 | Sustainable Transport |
| 25 | Picture of bus, pedestrian, and cyclist |
| 26 | I get around  Bus. Train. Tram. Car share. Walk. Ride. |

## Non-environmental Signs

### Safer Community

| **Sign Number** | **Sign Description** |
| --- | --- |
| 27 | The University of Melbourne  A safe campus is everybody’s business. |
| 28 | The University of Melbourne  If you think it’s wrong you are probably right. |
| 29 | Security notice  Do not leave bags or luggage unattended |
| 30 | Alcohol, drugs, tobacco, weapons free zone |
| 31 | Zero tolerance zone |

### Emergency

| **Sign Number** | **Sign Description** |
| --- | --- |
| 32 | Fire dept |
| 33 | Fire danger rating today |
| 34 | Police |
| 35 | 000 Emergency |
| 36 | Emergency calls dial…  000  Fire, Police, Ambulance  Police 131 444 |

### Hospital

| **Sign Number** | **Sign Description** |
| --- | --- |
| 37 | Hospital (blue sign with arrow) |
| 38 | Hospital (white cross on green background) |
| 39 | No text medical snake on a white asterisk on a blue background |
| 40 | Red cross on white background |
| 41 | Red cross on white background with blue frame |

### Construction

| **Sign Number** | **Sign Description** |
| --- | --- |
| 42 | No text: Construction worker on orange background |
| 43 | Danger  Construction site  Unauthorised persons keep out |
| 44 | This is a hard hat area  Safety helmets must be worn |
| 45 | Restricted area  Construction work in progress |
| 46 | Construction site  Keep out |

### Marine

| **Sign Number** | **Sign Description** |
| --- | --- |
| 47 | No text  Landing forbidden |
| 48 | No text  Anchoring forbidden |
| 49 | No text  Landing allowed |
| 50 | No text  Turning engine |
| 51 | Notice  No windsurfing |

## Additional signs only in Study 2

### Marine Wildlife

| **Sign Number** | **Sign Description** |
| --- | --- |
| 52 | This seal is being monitored  Please respect  Seals bite  Enjoy from 30 meters  Are protected wildlife  Leave alone |
| 53 | Attention  Seals have been using this beach to rest  Seals are protected wildlife  For your safety and the seal’s protection Do Not approach or feed the seal |
| 54 | Danger  Seals bite!  Seals are protected wildlife  For your safety and the seal’s protection DO NOT approach or feed the seal |
| 55 | Penalty $2,000 |
| 56 | Wild seal resting  Keep your distance |

### Other Animal Signs

| **Sign Number** | **Sign Description** |
| --- | --- |
| 57 | Do not touch animals |
| 58 | No text (Do not feed animals) |
| 59 | Keep wildlife wild  Our food is not healthy for wildlife  Never feed or approach them |
| 60 | Keep wildlife wild |
| 61 | Keep wildlife wild |

### Don’t touch signs

| **Sign Number** | **Sign Description** |
| --- | --- |
| 62 | Do not run |
| 63 | No running |
| 64 | Safety first  No cell phones while working |
| 65 | No text (No cell phones) |
| 66 | Notice  No cell phones beyond this point |

### New Environmental Signs

| **Sign Number** | **Sign Description** |
| --- | --- |
| 67 | Please don’t waste water  Protect the environment for our children |
| 68 | Every drop counts  Reduce your use |
| 69 | Do not waste water |
| 70 | No text  Throw paper in bin |
| 71 | Please don’t waste paper  Protect the environment for our children |
