# Supplementary material for: Signage as a tool for behavioral change: Direct and indirect routes to understanding the meaning of a sign
Source: PLoS One. 2017 Aug 30;12(8):e0182975. doi: 10.1371/journal.pone.0182975 (PMC5576639; doi:10.1371/journal.pone.0182975)
Supplement: S2 Table — (DOCX) [file pone.0182975.s002.docx]

# S2 Table. Descriptive Statistics for Perceived Effectiveness, Familiarity, and Clarity of Purpose for Study 1.

|  |  |  | Perceived Effectiveness | | Familiarity | | Clarity of Purpose | |
| --- | --- | --- | --- | --- | --- | --- | --- | --- |
| Sign Group |  | *n* | *M* | *SD* | *M* | *SD* | *M* | *SD* |
| Environmental Signs |  |  |  |  |  |  |  |  |
| Recycling |  | 5 | 72,91 | 5,86 | 1,51 | 1,21 | 0,99 | 0,03 |
| Paper Use |  | 5 | 65,26 | 7,92 | 0,71 | 0,91 | 0,98 | 0,03 |
| Water Use |  | 6 | 66,88 | 7,72 | 0,75 | 0,37 | 0,96 | 0,04 |
| Energy Use |  | 5 | 66,87 | 4,51 | 0,62 | 0,37 | 0,80 | 0,25 |
| Sustainable Transport |  | 5 | 54,94 | 6,48 | 0,44 | 0,50 | 0,62 | 0,27 |
| Non-environmental Signs |  |  |  |  |  |  |  |  |
| Safe Community Living |  | 5 | 69,47 | 9,95 | 1,26 | 0,60 | 0,78 | 0,17 |
| Emergency |  | 5 | 69,06 | 11,27 | 1,96 | 0,80 | 0,64 | 0,19 |
| Hospital |  | 5 | 55,40 | 13,49 | 1,72 | 0,74 | 0,83 | 0,23 |
| Construction |  | 5 | 77,86 | 12,02 | 2,32 | 0,36 | 0,45 | 0,07 |
| Marine Traffic |  | 5 | 38,65 | 22,90 | 0,21 | 0,36 | 0,53 | 0,16 |
| Total |  | 51 | 63,79 | 14,81 | 1,14 | 0,91 | 0,76 | 0,24 |
