# Supplementary material for: Signage as a tool for behavioral change: Direct and indirect routes to understanding the meaning of a sign
Source: PLoS One. 2017 Aug 30;12(8):e0182975. doi: 10.1371/journal.pone.0182975 (PMC5576639; doi:10.1371/journal.pone.0182975)
Supplement: S3 Table — (DOCX) [file pone.0182975.s003.docx]

# S3 Table. Descriptive Statistics for Perceived Effectiveness, Familiarity, and Clarity of Purpose for Study 2.

|  |  | Perceived Effectiveness | | Familiarity | | Clarity of Purpose | |
| --- | --- | --- | --- | --- | --- | --- | --- |
| Sign Group | *n* | *M* | *SD* | *M* | *SD* | *M* | *SD* |
| Environmental Signs |  |  |  |  |  |  |  |
| Recycling | 5 | 74,28 | 6,50 | 1,65 | 1,19 | 0,97 | 0,04 |
| Paper Use | 5 | 63,83 | 9,34 | 0,75 | 0,74 | 0,91 | 0,10 |
| Water Use | 6 | 63,34 | 9,56 | 0,77 | 0,31 | 0,91 | 0,04 |
| Energy Use | 5 | 67,43 | 8,63 | 0,80 | 0,33 | 0,71 | 0,29 |
| Sustainable Transport | 5 | 50,62 | 7,65 | 0,47 | 0,39 | 0,54 | 0,23 |
| Non-environmental Signs |  |  |  |  |  |  |  |
| Safe Community Living | 5 | 66,96 | 13,51 | 1,36 | 0,64 | 0,62 | 0,17 |
| Emergency | 5 | 72,63 | 13,66 | 2,14 | 0,88 | 0,65 | 0,15 |
| Hospital | 5 | 60,02 | 13,15 | 1,91 | 0,62 | 0,46 | 0,08 |
| Construction | 5 | 77,21 | 8,39 | 2,34 | 0,40 | 0,87 | 0,16 |
| Marine Traffic | 5 | 42,46 | 23,23 | 0,24 | 0,32 | 0,38 | 0,11 |
| Additional Signs for Study 2 |  |  |  |  |  |  |  |
| Marine Wildlife | 5 | 80,17 | 4,50 | 0,26 | 0,12 | 0,76 | 0,03 |
| Wildlife | 5 | 75,64 | 4,09 | 0,81 | 0,44 | 0,71 | 0,10 |
| Additional Safety Signs | 5 | 69,73 | 2,68 | 1,78 | 0,87 | 0,48 | 0,12 |
| Additional Environmental Signs | 5 | 68,44 | 4,85 | 1,29 | 0,86 | 0,94 | 0,02 |
| Total | 71 | 66,58 | 13,80 | 1,18 | 0,89 | 0,71 | 0,23 |
