# Supplementary material for: Signage as a tool for behavioral change: Direct and indirect routes to understanding the meaning of a sign
Source: PLoS One. 2017 Aug 30;12(8):e0182975. doi: 10.1371/journal.pone.0182975 (PMC5576639; doi:10.1371/journal.pone.0182975)
Supplement: S1 Text — (DOCX) [file pone.0182975.s004.docx]

# S1 Text. Assumptions of Multiple Regression.

We tested whether our data satisfy the general assumptions for multiple regression as reported in Kleinbaum [1], Field [2], and Pallant [3]. In both studies, the independent variables showed some correlations with the dependent variables (around *r* = .3), but the independent variables did not correlate with each other. Furthermore, tolerance statistics and VIF (variance inflation factor) were around the recommended values and thus did not suggest the possibility of multicollinearity (tolerance statistics were around .9 and VIF around 1.0). Inspection of the scatterplot and histogram showed a normal distribution of the residuals, and thus an important assumption about the independence of residuals was met. This conclusion was supported by the Durbin-Watson statistic showing a value close to 2, which further supports the assumption of independent errors.

We acknowledge that our data violated the assumption of normality. In both studies, the dependent variable was significantly negatively skewed and showed significant positive kurtosis. Kolmogorov-Smirnov test of normality was marginally significant for Study1 (*p* = .06) and significant for Study 2 (*p* < .05). The independent variables were not skewed, but Clarity of Purpose showed significant negative kurtosis. Kolmogorov-Smirnov test of normality was significant for both predictors supporting the violation of normality. According to Stevens [4], our sample size was sufficient to fulfil the criteria for multiple regression and several authors [1, 5, 6] stated assumption of normality as less important for multiple regression as long as the independence of residuals is present, which was the case.

**References**

1. Kleinbaum DG. Applied regression analysis and other multivariable methods. 4th ed. Belmont, CA: Brooks/Cole 2008.

2. Field AP. Discovering statistics using SPSS. 3rd ed. London: SAGE; 2009.

3. Pallant J. SPSS survival manual: A step by step guide to data analysis using SPSS. 4th ed. Crows Nest, N.S.W: Allen & Unwin; 2011.

4. Stevens J. Applied multivariate statistics for the social sciences. 3rd ed. Mahwah, N.J: Lawrence Erlbaum Associates; 1996.

5. Lumley T, Diehr P, Emerson S, Chen L. The importance of the normality assumption in large public health data sets. Annual Review Of Public Health. 2002;23:151-69. doi: 10.1146/annurev.publhealth.23.100901.140546. PubMed PMID: 11910059.

6. Stuart A, Ord JK, Arnold S. Kendall's advanced theory of statistics. Vol. 2A. New York: Oxford University Press; 1999.
